# Impact of Traumatic Dental Injuries on Oral Health‐Related Quality of Life Among Primary School Children in Dar es Salaam

**DOI:** 10.1002/cre2.70105

**Published:** 2025-02-23

**Authors:** Gloria Leo, Kasusu Nyamuryekung'e, Febronia Kokulengya Kahabuka

**Affiliations:** ^1^ Department of Dental Services Muhimbili National Hospital Dar es Salaam Tanzania; ^2^ Department of Orthodontics, Pedodontics and Community Dentistry, School of Dentistry Muhimbili University of Health and Allied Sciences Dar es Salaam Tanzania

**Keywords:** child, oral health, quality of life, Tanzania, tooth injuries

## Abstract

**Objectives:**

Traumatic dental injuries (TDIs) are a significant concern in pediatric populations, affecting children's oral health‐related quality of life (OHRQoL) and overall development. This study aims to examine TDI prevalence, types, risk factors, and impacts on OHRQoL among Tanzanian school children.

**Materials and Methods:**

Employing a cross‐sectional analytical approach, the study assessed TDIs in school children aged 8–10 in Ilala district, Tanzania, using a multistage cluster sampling technique. Data collection employed structured questionnaires and clinical examinations, applying Ellis and Davey's classification and the Child Perception Questionnaire (CPQ 8–10) for TDIs and OHRQoL assessments, respectively. Statistical analyses included frequency distributions, chi‐square, Mann–Whitney and Kruskal–Wallis tests, and binary logistic regression.

**Results:**

Among 501 participants, 25.1% reported previous oral trauma with a TDI prevalence of 37.5%. Crown fractures were the most common injury (79.8%), with significant associations found between TDI prevalence and inadequate lip coverage (*p* = 0.037) and overjet (*p* < 0.001). Over 60% experienced an oral impact, notably in oral symptoms (52.5%), emotional well‐being (25.1%), and functional limitation (22.8%) domains. Children with TDIs showed higher OHRQoL impacts on emotional and social well‐being domains.

**Conclusions:**

The high prevalence of TDIs among Tanzanian school children significantly impacts their OHRQoL, emphasizing the need for comprehensive pediatric dental care approaches that encompass preventive strategies and address the multifaceted aspects of oral health.

## 1 Introduction

Traumatic dental injuries (TDIs) constitute a significant concern within pediatric populations globally, presenting not only as a prevalent clinical occurrence but also as a complex social and public health challenge. The occurrence of TDIs in children and adolescents spans a broad spectrum, with reported prevalence rates in permanent teeth ranging from 9.3% to 59% and in deciduous teeth from 14.2% to 27%, highlighting the pervasive nature of these injuries across different dental stages (Petti et al. 2018). In recent years, a wide range of TDI prevalence has been reported across different populations, with Silva‐Oliveira et al. (2018) reporting a prevalence of 29.4% among 12‐year‐olds, Al‐Ansari and Nazir (2020) reporting 39.5% among 12–15‐year‐old children whereas Patnana et al. (2021) reported through a systematic review an overall prevalence of 24.2% in primary teeth (Silva‐Oliveira et al. 2018; Patnana et al. 2021; Al‐Ansari and Nazir 2020). A notable gender disparity exists, with a higher frequency of TDIs observed in male children, attributed to their increased engagement in physical activities and sports, particularly at ages where risk‐taking behaviors are more common (Magno et al. 2020; Sulieman and Awooda 2018).

The etiology of TDIs is multifaceted, predominantly stemming from unintentional falls, sports‐related incidents, and other physical activities, underscoring the heightened vulnerability of children during key developmental phases such as early mobility and active play (Lin and Naidoo 2008). The consequences of untreated or inadequately managed TDIs extend beyond immediate physical trauma, potentially impairing oral health‐related quality of life (OHRQoL) and affecting various aspects of child development, including functional capacity, social interactions, and psychological well‐being.

Considering the sizeable impact of TDIs on OHRQoL, there has been a growing emphasis on utilizing comprehensive evaluation tools, such as the Child Perception Questionnaire (CPQ), to assess the broader implications of dental trauma on children's health and quality of life. These tools have demonstrated efficacy in capturing the multifaceted consequences of TDIs, thereby facilitating a more holistic approach to understanding and addressing the needs of affected children.

Several studies have indicated a negative impact on overall OHRQoL in children with TDIs aged between 8 and 14 years, including poor physical function, poor social‐psychological life, and pain (Jokovic et al. 2003). Children have reported to have poor social interactions and faced rejections in groups and social functions by other children, experienced anxiety, refused to attend school fearing mocking from other children, as well as having difficulty in chewing and pain (Abanto et al. 2011; Goursand et al. 2008).

Evidence from systematic reviews reveals that TDIs cause a negative impact on OHRQoL based on the overall Early Childhood Oral Health Impact Scale – ECOHIS (Borges et al. 2017), which was also supported by Antunes et al. (2020) who reported that the impact of TDI on OHRQoL in children under age 10 was only significant in the symptom domain using ECOHIS (Antunes et al. 2020; Borges et al. 2017). A systematic review by Das et al. (2022) detailed that TDI of permanent teeth strongly influences the OHRQoL of children and adolescents.

In the African context, the prevalence of TDIs among children and adolescents exhibits significant variability. Studies conducted across different regions within the continent reveal a TDI prevalence ranging from 6.4% among South African school children, 9.1% among Sudanese school children to 21% in Tanzanian youths (Naidoo et al. 2009; Kahabuka et al. 2001). A previous study among Tanzanian children reported the most common dental trauma to be enamel fracture (67%), followed by enamel‐dentin fracture without pulp involvement (26%) (Kahabuka et al. 2001). Furthermore, male sex, increased overjet, and lip incompetence have been identified as risk factors of trauma to maxillary incisors in the same setting (Kahabuka and Mugonzibwa 2009). However, considering that these studies were conducted more than a decade ago, it is possible that an epidemiological shift in the landscape has occurred.

Despite the extensive body of research on TDIs, gaps remain in our understanding of the current prevalence and impact of these injuries on children's quality of life. This is particularly relevant in regions where dental health may not be prioritized, such as in developing countries. This study aims to determine the prevalence, types, and risk factors associated with TDIs among Tanzanian school children. Furthermore, this research seeks to explore the impact of TDIs on the OHRQoL of this population, contributing valuable insights to inform targeted prevention and management strategies.

## 2 Materials and Methods

This observational study has been written according to Preferred Reporting items for Observational studies in Endodontics (PROBE) 2023 guidelines (Nagendrababu et al. 2022).

A cross‐sectional analytical design was employed to assess the prevalence and impact of TDIs among primary school children aged 8–10 years. The study was conducted in both public and private schools within the Ilala district of the Dar es Salaam region, targeting an age group identified as highly susceptible to various types of dental injuries. The district has a total of 120 public and 102 private primary schools, providing a sample of children from varied socioeconomic backgrounds.

The sample size was determined using a formula for population frequency, accounting for an expected outcome factor of 20%, a 5% confidence limit, and a design effect of 2 to account for a complex sampling strategy. This calculation, based on assumptions from a hypothetical population of 1,000,000, yielded a sample size of 492 children, ensuring a 95% confidence level. Children aged 8–10 years were eligible for inclusion, while those with severe mental disabilities impairing communication were excluded from the study.

A multistage non‐proportional cluster sampling was implemented to select schools and pupils that were included in the study. Ilala district has 34 wards, with variation in number of private and public schools. Seven Wards without both private and public schools were excluded from selection list. Thereafter, 20% of the remaining wards (27) were randomly selected, making a total of 6 wards. Each selected ward was an independent stratum. In each stratum, two schools, a public and a private one, were randomly selected, making a total of 12 sampled schools from the district. Children were randomly selected using class registers as a sampling frame whereby between 41 and 43 children aged 8, 9, and 10 years were randomly selected to form the sample size from each school.

Data were gathered using a structured questionnaire designed to collect sociodemographic details, causes of trauma, and risk factors. The questionnaire also included items adapted from the Ellis and Davey trauma classification for TDI assessment and the CPQ 8–10 to evaluate the OHRQoL in children.

The clinical examiner (G.L.) had undergone training through a blended approach, utilizing both theoretical and visual (photographs) components on identifying types of dental trauma according to Ellis and Davey trauma classification from a previously calibrated expert (F.K.) on TDIs types. The clinical examiner also underwent 1‐week additional training program on how to utilize the CPQ 8–10 questionnaire. A pilot study involving 50 participants from a nonparticipating school in Ilala was conducted to validate the data collection instrument. Adjustments were made based on response rates, questionnaire completion frequencies, and average completion times.

Participants' sociodemographic information and clinical data were obtained through interviews and examinations. Clinical data on TDIs for the permanent dentition of each participant were collected through examination and recorded according to Ellis and Davey's trauma classification method. Clinical examination for TDIs was done whilst participants were seated upright on a chair, facing a window or any other source of natural light. The examiner wore gloves, used naked eyes, and was aided by a mouth mirror and probe. Assessment of the dentition for TDIs was done after the examiner dried the teeth with sterile gauze and controlled moisture using cotton rolls. Examination was done systematically, starting from the first quadrant till the fourth quadrant. The assessment of the dentition was conducted in pairs, whereby the examiner reported the findings verbally for the recorder to note down in the questionnaire of the respective participant.

A graduated William's probe was utilized for over‐jet assessment. Over‐jet was measured in mm, with the tip of the probe touching the Buccal surface of the upper anterior teeth from the incisor cusp to the point corresponding to the level of the lower incisor teeth. When the distance recorded was below 4 mm, it was documented as normal, and if 4 mm or above, it was indicated as increased overjet. Lip competence was assessed and measured as absent when the lips were in normal position without any restriction on mouth closing at complete rest. In case of any observed restriction of mouth closing, it was indicated as an incompetent lip.

OHRQoL was assessed by the CPQ 8–10 questionnaire, a standard subjective tool for assessment of OHRQoL, consisting of four domains: Oral symptoms, function limitation, emotional well‐being, and social well‐being (Jokovic et al. 2003). A total of 25 questions were assessed, whereby oral symptoms, function limitation, and emotional well‐being domains each consisted of 5 questions, while the social well‐being domain consisted of 10 questions. In each of the questions, participants responded according to their experience as; never, once or twice, sometimes, often, and daily.

Sociodemographic and clinical data were analyzed as categorical variables, presented through frequencies and percentages. The overall prevalence of TDIs was calculated as a proportion of participants that had at least one TDI on clinical examination irrespective of type, and chi‐square tests were used to examine associations between sociodemographic characteristics, risk factors, and TDIs.

OHRQoL using the CPQ 8–10 tool was first presented as frequencies and percentages of those responding to have experienced the stated impacts according to the questionnaire. All those who reported “never” to have experienced any of the statements were considered to not have had any impact on their quality of life. Those reporting to have had the stated experience “once or twice, sometimes, often or daily” were categorized as having had an impact on their quality of life. OHRQoL data were analyzed using frequencies, percentages across CPQ 8–10 domains, and associations between TDIs and CPQ domains examined using Mann–Whitney or Kruskal–Wallis tests.

Binary logistic regression analysis was used to explore the associations of selected SES and epidemiological factors with reported OHRQoL impacts. The logistic regression models were used to study the effects of the predictor variables, while having the effects of other variables in the model simultaneously controlled.

The study received approval from the Muhimbili University of Health and Allied Sciences Institutional Review Board (IRB) through letter Ref. No. DA.282/298/01.C/. Permission to conduct the study from relevant local and school authorities in Ilala was also obtained. Informed consent was obtained from the parents or guardians of participants, with assent from the children. Confidentiality was maintained using codes, and participants had the right to withdraw at any time from the study.

## 3 Results

A total of 501 primary school students participated in this study. More than half (59.7%) were ten‐year‐old, female (58.3%) and about two‐thirds (60.3%) were attending public schools. About a quarter (25.1%) of the students reported to have sustained oral trauma previously. Almost all (90%) of the pupils who reported to have sustained oral trauma had either fallen (61.1%) or been struck by an object (27.0%) (Table 1).

**Table 1:** Characteristics of the participants.

Variable	Frequency	Percent
Age		
8 years	73	14.6
9 years	129	25.7
10 years	299	59.7
Sex		
Male	209	41.7
Female	292	58.3
Type of school		
Public	302	60.3
Private	199	39.7
Previous oral trauma		
Yes	126	25.1
No	375	74.9
Causes of oral trauma (*n* = 126)		
Fall	77	61.1
Physical assault	2	1.6
Struck by an object	34	27.0
Collision with another child	4	3.2
Motor traffic accident	2	1.6
Others	3	2.4
Don't remember	4	3.2

The prevalence of TDIs in the studied population was 37.5%. The most common types of injuries were simple fractures of the crown (79.8%) and extensive fractures of the crown, involving considerable dentin, but not the pulp (34.0%). A small proportion (2.7%) had complete tooth loss resulting from the sustained trauma (Table 2).

**Table 2:** The distribution of traumatic dental injuries among participants.

Variable	Frequency	Percent
Overall prevalence of TDIs		
Any type of traumatic dental injury	188	37.5
Prevalence of specific types of TDIs		
Simple fracture of the crown: involving little or no dentin	150	79.8
Extensive fracture of the crown: involving considerable dentin but not the pulp	64	34.0
Tooth lost because of trauma	5	2.7
Extensive fracture of the crown: involving considerable dentin and exposing the dental pulp	0	0
The traumatized tooth which becomes non: vital with or without loss of crown structure	0	0
Displacement of the tooth: without fracture of crown or root	0	0
Fracture of the crown en masse (all together) and its displacement	0	0

No association of TDIs by age, sex, or type of school was detected. Nevertheless, a significantly higher proportion of participants reporting to have had a previous history of oral trauma (57.1%) were clinically assessed to have a TDI compared to those with a negative history of oral trauma (30.9%). Further, participants clinically assessed with inadequate lip coverage (*p* = 0.037) and overjet of more than 4 mm (*p* < 0.001) had higher prevalence of TDI compared to their counterparts (Table 3).

**Table 3:** The association between demographic characteristics, risk factors, and traumatic dental injuries among participants.

	Occurrence of TDIs		
Variable	TDIs Absent, *n* (%)	TDIs present, *n* (%)	(χ^2^) *p* value
Age (in years)			
8	42 (57.5)	31 (42.5)	(0.912) 0.634
9	81 (62.8)	48 (37.2)	
10	190 (63.5)	109 (36.5)	
Sex			
Male	138 (66.0)	71 (34.0)	(1.932) 0.165
Female	175 (59.9)	117 (40.1)	
Type of school			
Public	197 (65.2)	105 (34.8)	(2.465) 0.166
Private	116 (58.3)	83 (41.7)	
Previous oral trauma			
Yes	54 (42.9)	72 (57.1)	(27.635) < 0.001
No	259 (69.1)	116 (30.9)	
Lip Coverage			
Adequate	226 (65.5)	119 (34.5)	(4.345) 0.037
Inadequate	87 (55.8)	69 (44.2)	
Over‐jet (mm)			
0–4 mm	174 (75.3)	57 (24.7)	(30.188) < 0.001
More than 4 mm	139 (51.5)	131 (48.5)	

Regarding the CPQ variables, 39% of the participants did not report an oral impact in any of the assessed domains. However, most respondents reported to have an oral impact in the oral symptom (52.5%), followed by emotional well‐being (25.1%), functional limitation (22.8%), and social well‐being (13.8%) domains.

An assessment of variation in CPQ domains by demographic, clinical findings, and history of trauma among the patients was conducted. Social well‐being scores were observed to differ among participants by age (*p* = 0.024). Also, females had higher median oral symptom (*p* = 0.046) and emotional well‐being (*p* = 0.006) scores than males. Those with a positive history of oral trauma had higher median oral symptoms (*p* = 0.016), functional limitation (*p* = 0.041), emotional well‐being (*p* = 0.004), and social well‐being (*p* = 0.010) scores than pupils without previous trauma. Furthermore, pupils with TDIs had a higher median emotional and social well‐being scores than those without TDIs, *p*= 0.022 and 0.034, respectively (Table 4).

**Table 4:** Association of demographic, history of trauma, over‐jet, and traumatic dental injury by Oral Health‐Related Quality of life scores (Median scores and *p* values according to Kruskal–Wallis and Mann–Whitney tests).

Variable	Oral symptoms		Functional limitation		Emotional well‐being		Social well‐being		Overall	
	Median (IQR)	*p* value	Median (IQR)	*p* value	Median (IQR)	*p* value	Median (IQR)	*p* value	Median (IQR)	*p* value
Age (years)										
8	0.0 (2.0)	0.309	0.0 (1.0)	0.305	0.0 (0.0)	0.145	0.0 (0.0)	**0.024**	1.0 (4.5)	0.104
9	0.0 (2.0)		0.0 (0.0)		0.0 (0.0)		0.0 (0.0)		1.0 (3.0)	
10	1.0 (2.0)		0.0 (0.0)		0.0 (1.0)		0.0 (0.0)		1.0 (5.0)	
Sex										
Male	0.0 (2.0)	**0.046**	0.0 (0.0)	0.368	0.0 (0.0)	**0.006**	0.0 (0.0)	0.510	1.0 (3.0)	**0.012**
Female	1.0 (3.0)		0.0 (0.0)		0.0 (1.0)		0.0 (0.0)		1.0 (5.0)	
Type of school										
Public	1.0 (2.0)	0.413	0.0 (0.0)	0.453	0.0 (1.0)	0.343	0.0 (0.0)	0.538	1.0 (5.0)	0.372
Private	1.0 (2.0)		0.0 (0.0)		0.0 (0.0)		0.0 (0.0)		1.0 (4.0)	
Previous oral trauma										
Yes	1.0 (3.0)	**0.016**	0.0 (1.0)	**0.041**	0.0 (1.0)	**0.004**	0.0 (0.0)	**0.010**	2.0 (6.0)	**0.001**
No	0.0 (2.0)		0.0 (0.0)		0.0 (0.0)		0.0 (0.0)		1.0 (4.0)	
Lip coverage										
Adequate	1.0 (2.0)	0.169	0.0 (0.0)	0.330	0.0 (0.0)	0.185	0.0 (0.0)	0.522	1.0 (4.0)	0.847
Inadequate	0.0 (2.0)		0.0 (1.0)		0.0 (1.0)		0.0 (0.0)		1.0 (4.0)	
Overjet (mm)										
0–4 mm	1.0 (3.0)	0.319	0.0 (0.0)	0.635	0.0 (0.0)	0.427	0.0 (0.0)	0.593	1.0 (4.0)	0.883
More than 4 mm	1.0 (2.0)		0.0 (0.0)		0.0 (1.0)		0.0 (0.0)		1.0 (4.0)	
TDIs										
Present	1.0 (2.0)	0.535	0.0 (0.0)	0.922	0.0 (1.0)	**0.022**	0.0 (0.0)	**0.034**	1.0 (5.0)	0.414
Absent	1.0 (2.0)		0.0 (0.0)		0.0 (0.0)		0.0 (0.0)		1.0 (4.0)	

*Note:* The bolded numbers highlight *p *values that are significant (<0.05).

Direct logistic regression was conducted to evaluate the impact of sex, clinically observed TDI, and a history of previous oral trauma on the likelihood of reporting an OHRQoL impact, as measured by the CPQ 8–10. The full model, which included all predictors, was statistically significant, χ² (3, *N* = 501) = 15.06, *p* < 0.01, indicating the model effectively distinguished between respondents with and without an OHRQoL impact. The model explained between 3% (Cox and Snell *R* square) and 4% (Nagelkerke *R* square) of the variance in OHRQoL impact, correctly classifying 61.5% of cases. Two independent variables—sex and previous oral trauma—made a unique and statistically significant contribution. The strongest predictor of reporting an OHRQoL impact was a history of previous oral trauma, with an odds ratio of 2.06, indicating that respondents with an OHRQoL impact were twice as likely to report previous oral trauma compared to those without an OHRQoL impact, controlling for other factors. The odds ratio for sex was 1.54, indicating that females were 1.5 times more likely to report an OHRQoL impact, after adjusting for other variables (Table 5).

**Table 5:** Logistic regression predicting likelihood of having an OHRQoL impact.

		B	S.E.	Wald	df	*P* value	Odds ratio	95% C.I. for odds ratio	
								Lower	Upper
Sex		0.428	0.188	5.201	1	0.023	1.535	1.062	2.218
TDI		−0.105	0.198	0.278	1	0.598	0.901	0.611	1.328
Previous Oral Trauma		0.723	0.232	9.699	1	0.002	2.061	1.307	3.248
Constant		0.058	0.160	0.130	1	0.719	1.059		

## 4 Discussion

The findings from this study reveal a notable prevalence of TDIs among Tanzanian children, with more than one‐third of the participants having experienced some form of TDI. This observed prevalence aligns with global findings, which have documented varying TDI rates among school‐aged children, often influenced by geographical, socioeconomic, and cultural factors (Petti et al. 2018; Glendor 2008).

The predominance of simple crown fractures, constituting nearly 80% of the TDIs observed, is consistent with the literature, suggesting that enamel fractures are the most common type of TDIs in children due to the resilience of young dental tissues and the nature of impacts typically encountered in childhood (DiAngelis et al. 2012; Marcenes et al. 2001). The occurrence of extensive crown fractures involving considerable dentin but not the pulp in 34.0% of cases indicates a significant force on impact, likely necessitating professional dental intervention to prevent further complications such as pulpitis or infection (Andersson et al. 2012). This type of fracture highlights the potential severity of TDIs even in cases where the pulp is not initially compromised and underscores the need for prompt dental assessment following trauma. The finding that approximately one‐quarter of the children had previously sustained trauma around the oral cavity, with the majority being due to falls, is reflective of the age group's heightened risk due to developmental factors such as active play, sports participation, and less developed motor coordination (Bourguignon and Sigurdsson 2009; Lam 2016).

The association of Inadequate lip coverage with TDIs points to the protective role that soft tissues play in cushioning the impact on teeth in the event of trauma. The lips, especially when competent and able to cover the anterior teeth, can absorb and distribute forces that might otherwise directly fracture or displace teeth. The significance of lip coverage in the context of dental trauma has been corroborated by several studies, which suggest that inadequate lip coverage is a risk factor for TDIs, particularly in activities where facial impacts are likely (Petti et al. 2018; Kramer et al. 2013). The association of an overjet greater than 4 mm with an increased risk of TDIs is consistent with the mechanical disadvantage posed by protruding anterior teeth. An increased overjet makes the anterior teeth more susceptible to direct trauma, as they are the first point of contact in many injury scenarios. This finding is supported by other studies that have consistently identified increased overjet as a significant risk factor for TDIs in children (Kahabuka and Mugonzibwa 2009; DiAngelis et al. 2012; Marcenes et al. 2001). Collectively, our findings support the need for routine dental evaluations to assess and document risk factors such as overjet and lip coverage, allowing for early identification of children who may benefit from preventive advice or orthodontic interventions to mitigate the risk of TDIs.

The findings from our study regarding the CPQ variables offer insightful perspectives on the OHRQoL among 8‐ and 10‐year‐old children. It is noteworthy that more than a third of our participants did not perceive any negative oral health impacts across the assessed domains. This suggests a relatively good OHRQoL in a significant segment of this demographic. Conversely, over half of the participants (52.5%) reported experiencing negative impacts predominantly in the “oral symptom” domain, indicating that physical symptoms related to oral health issues are the most perceptible and concerning for children within this age group. This high prevalence of oral symptoms aligns with findings from previous studies, which have also highlighted physical discomfort as a primary concern among children with oral health problems (Jokovic et al. 2003; Locker 2007). The prominence of oral symptoms such as pain, sensitivity, and discomfort highlights the need for focused dental care and preventive measures targeting common pediatric oral health issues like dental caries and gingivitis.

The reported impacts on “emotional well‐being” and “functional limitations” domains, cited by 25.1% and 22.8% of participants, respectively, reflect the broader psychosocial consequences of oral health issues. These findings resonate with those of Kumar et al who observed that dental problems in children could significantly affect their emotional state and daily functions, such as eating and speaking (Kumar et al. 2014). The emotional distress associated with oral health problems, including fear, embarrassment, and frustration, can notably affect a child's self‐esteem and social interactions (Abanto et al. 2011; Goursand et al. 2008). Interestingly, the “social well‐being” domain was the least affected, with only 13.8% of participants reporting impacts. This lower level of reported social impact might suggest that, within this age group, the social ramifications of oral health issues are less pronounced or perceived compared to physical and emotional aspects. However, this does not undermine the significance of even minimal impacts on social well‐being, as social interactions and peer acceptance are crucial during this developmental stage (Foster Page et al. 2005; Paula et al. 2012).

The results of comparative and regression tests conducted in this study reveal significant gender disparities in OHRQoL among the children surveyed, with females reporting higher median scores in oral symptoms, emotional well‐being, and overall quality of life compared to their male counterparts. These findings are consistent with existing literature, which suggests that females often report greater impacts of oral health conditions on their quality of life than males (Locker 2007; Piovesan et al. 2011). This discrepancy may be attributed to gender differences in perception, reporting of pain and discomfort, and societal roles and expectations that influence the psychosocial impact of oral health issues (Thomson and Locker 2001; Slade 2001).

Moreover, the study highlights the lasting impact of previous oral trauma on children's OHRQoL. Children with a history of oral trauma exhibited significantly higher median scores across all domains—oral symptoms, functional limitation, emotional, and social well‐being—and strongly predicted OHRQoL impacts compared to those without such a history. This suggests that the effects of oral trauma extend beyond the immediate physical injuries, contributing to long‐term functional and psychosocial challenges (Traebert et al. 2003; Oliveira et al. 2015). The elevated scores in the emotional and social well‐being domains emphasize the profound influence of past traumatic experiences on children's psychological and social interactions, potentially due to lingering pain, appearance‐related concerns, and the traumatic memory itself (Krisdapong et al. 2009; Nagendrababu et al. 2023).

Our study's limitations, including its cross‐sectional nature and age‐specific focus, may have led to an underrepresentation of the full spectrum of TDIs. Also, the usage of one examiner may have introduced bias during clinical examinations although care was taken to carefully calibrate the examiner before the conduct of this study. Furthermore, the reliance on clinical examinations potentially overlooked resolved or treated injuries, suggesting a need for comprehensive historical data collection in future research.

Our findings underscore the urgent need for public health initiatives focused on oral health education, injury prevention, and the promotion of safe play environments in schools for children. Policies supporting the integration of oral health programs into school health services could be critical in addressing the observed TDI prevalence (Jain et al. 2018). Furthermore, dental practitioners should be aware of the high incidence of TDIs in this age group and the common types of injuries encountered. This awareness can inform clinical practice, particularly in terms of preventive advice given to parents and children, and in enhancing the preparedness of dental professionals to manage such injuries effectively (Zaror et al. 2023). Lastly, we emphasize the importance of comprehensive care following oral trauma, which should address the physical injuries as well as consider potential long‐term emotional and social impacts.

The study's findings highlight the significant and multifaceted impacts of oral health issues, particularly TDIs, on children's quality of life, with notable differences observed based on history of oral trauma. These findings emphasize the need for establishing comprehensive dental health programs within schools. These should include education on oral hygiene, promote wearing protective gear during sports, and first‐aid measures for dental injuries as important measures in this setting.

## Author Contributions

Gloria Leo conceived, designed, and conducted the study, data analysis and interpretation, and drafting the manuscript. Kasusu Nyamuryekung'e participated in the design of the study, composition of the study tools, supervision of the research process, data analysis and interpretation, and critical revision of the manuscript. Febronia Kokulengya Kahabuka participated in study design, composition of the study tool, supervision of the research process and critical review of the manuscript.

## Conflicts of Interest

The authors declare no conflicts of interest.

## Data Availability

Data used for this study is available from the authors upon reasonable request.
